# Arrowhead (*Sagittaria cuneata*) as a bioindicator of nitrogen and phosphorus for prairie streams and wetlands

**DOI:** 10.1007/s11273-017-9576-5

**Published:** 2017-09-27

**Authors:** Katherine M. Standen, Patricia A. Chambers, Joseph M. Culp

**Affiliations:** 1 Biology Department and Canadian Rivers Institute, University of New Brunswick, PO Box 4400, Fredericton, NB E3B 5A3 Canada; 2 Environment and Climate Change Canada, 867 Lakeshore Rd, PO Box 5050, Burlington, ON L7R 4A6 Canada; 3 Environment and Climate Change Canada, University of New Brunswick, PO Box 4400, Fredericton, NB E3B 5A3 Canada

**Keywords:** Arrowhead, *Sagittaria*, Bioindicator, Nitrogen, Phosphorus, Great Plains

## Abstract

The emergent aquatic plant, *Sagittaria cuneata,* is an easily-identified and commonly-found species in the Great Plains region of North America and has the potential to be a bioindicator of nitrogen (N) and phosphorus (P) because of its previously-identified leaf plasticity in response to nutrient conditions. To identify associations between leaf morphology and soil and water nutrients, we conducted: (1) a 10-week controlled experiment in which plants were grown in nutrient-enriched sediment, nutrient-enriched water, or unamended control trials, and (2) a field study where emergent leaves were collected from 15 streams of varying nutrient concentrations. Plants grown in experimentally enriched sediment were more productive than those grown in enriched water or control conditions: they produced more emergent leaves and tubers, had a larger final biomass and height, and developed emergent leaves that showed a consistent increase in size and unique change in shape over time. Emergent leaves collected from field plants also showed significant variability of leaf traits; however, this variability occurred at all scales of replication (leaf, plant, quadrat, and site), with linear mixed effects modelling indicating that random chance was likely driving this variability. Although sediment nutrients were crucial to successful growth of *S. cuneata* under controlled conditions, the high variability in leaf morphology under field conditions (likely due to large natural variability at the species, population, and individual scale) make leaf plasticity of *S. cuneata* unsuitable as a bioindicator. Our results emphasize the need to quantify within and among plant variation in leaf morphology (and to clarify sampling methods) for the many taxa of aquatic macrophytes that are phenotypically plastic and notoriously difficult to classify.

**Electronic supplementary material:**

The online version of this article (doi:10.1007/s11273-017-9576-5) contains supplementary material, which is available to authorized users.

## Introduction

Bioindicators are common species, or groups of species, with easily identifiable features that exhibit plastic responses along gradients in one or more environmental variables (Holt and Miller 2011). Changes in species morphology, behavior, and physiology, presence or absence of taxa in a community, as well as the structure of the entire biotic community have all been used as indicators of stressors, both anthropogenic (pollution, land use changes) and natural (drought, flooding, etc.) (e.g., Richards and Ivey 2004). In streams (e.g., Clements and Carlisle 2000) and wetlands (e.g., Sharma and Rawat 2009), bioindicators have often been used to detect changes in water quality, such as eutrophication caused by excess nutrient inputs. Although the benthic macroinvertebrate (BMI) community has been the most commonly used taxa for assessment of water conditions (Hodkinson and Jackson 2005), aquatic macrophytes possess many traits characteristic of bioindicators: they are sessile, easily sampled and identified to genus (and often species), and respond to environmental stressors (e.g., nutrients, light, substrate texture, etc.) at the individual, population, and assemblage level. Nevertheless, compared to BMIs, relatively few studies have explored the use of macrophytes as bioindicators of environmental condition (e.g., Carbiener et al. 1990; Tremp and Kohler 1995; Robach et al. 1996; Demars and Harper 1998; Thiébaut and Muller 1999; Richards and Ivey 2004; Haury et al. 2006; Ceschin et al. 2010), especially in our region.

The aquatic macrophyte genus *Sagittaria* (Alismataceae) has the potential to be a useful bioindicator of nutrient status because of its easily identifiable characteristics, known plastic leaf morphology, and occurrence in and tolerance of a range of environmental conditions. *Sagittaria* is a submersed or emergent genus with extremely variable leaf size and shape (Arber 1920; Fernald 1950; Sculthorpe 1967): the emergent leaf blades range from lanceolate (lance-shaped) to sagittate (arrow-shaped) while the submerged leaves may be subulate (awl-shaped) or ribbon-like. Environmental conditions have been shown to influence leaf size and shape in this genus (e.g., Wooten 1986; Dorken and Barrett 2004; Richards and Ivey 2004). For example, Richards and Ivey (2004) found that *S. lancifolia* plants grown in a high P solution (1 mM) produced wider emergent leaves, more leaves per plant, and were larger overall than plants grown in a low P solution (10 µM), suggesting that higher P concentrations increased plant size and leaf width in *S. lancifolia*. Similarly, Dorken and Barrett (2004) noted that emergent leaf size of *S. latifolia* increased with fertilizer addition in both di- and monoecious populations. Water depth has also been found to influence leaf size, where increasing depth was associated with decreased leaf size as a result of energy being directed to enhance petiole length at the expense of leaf size (Wooten 1986). These predictable changes in leaf size with environmental conditions suggest that the genus *Sagittaria* may be a useful bioindicator of environmental stressors.

*Sagittaria cuneata* (Sheldon) is a widespread species across North America (Crow and Hellquist 2000) and is particularly common in parts of the Great Plains region, such as the Red River Valley in Manitoba, Canada. Here, initial conversion of native grassland to agricultural cropland or pasture, as well as present-day agricultural activities and urbanization, has led to inputs of pollutants to rivers, lakes, and wetlands, particularly sediment-bound and dissolved forms of nitrogen (N) and phosphorus (P) but also pesticides and heavy metals (Hall et al. 1999; Dodds et al. 2004; Donald et al. 2007). The Great Plains is considered an endangered biome (Samson and Knopf 1994), with many waterbodies at risk as a result of problems associated with agriculture and urbanization, including pollution, hydrologic disturbance, modifications of riparian zone, and channelization (Dodds et al. 2004). These changes are detrimental to ecological functioning and have led to changes in in-stream nutrient cycling that have impaired downstream water quality (Dodds et al. 2004). Within the Great Plains region of North America, agriculturally-derived nutrient loading to the Canada-USA transboundary Red River is particularly high (Environment Canada and Manitoba Water Stewardship 2011), prompting bioindicator research in order to quickly and accurately diagnose nutrient condition of streams and wetlands in this agriculturally-dominated watershed.

The aim of this study was to determine the association between leaf morphology of *Sagittaria cuneata* (Alismataceae) and nutrients found in both water and sediment as a first step towards development of a bioindicator of ecosystem nutrient status. Sediments have been previously shown to provide the majority of nutrients to aquatic plants (Barko and Smart 1980; Carignan and Kalff 1980; Huebert and Gorham 1983); however, the water column can also provide much of the required nutrients (Chambers 1987; Robach et al. 1996; Pelton et al. 1998) especially when the ratio of sediment to water nutrient availability is low (Rattray et al. 1991). We therefore conducted two studies: (1) a controlled experiment in which plants were grown in either standardized low-nutrient water or sediment, each independently supplemented with N or P and (2) a field study to determine variability in leaf morphology in relation to in situ water and sediment nutrient concentrations. These studies allowed us to establish, first, whether *S. cuneata* biomass and leaf morphology was primarily influenced by sediment or water nutrients in the absence of confounding effects (e.g., variability in incident light, sediment composition, water depth, water clarity, and stream velocity) and, second, to quantify patterns of variation in leaf characteristics within and among natural populations of *S. cuneata* in relation to nutrient conditions of streams and wetlands in the Red River Valley of Manitoba, Canada, and more widely in the North American Great Plains.

## Methods

### Experimental study

Tubers (i.e., nutrient-filled, clonal structures produced on underground rhizomes) were collected from two tributaries with populations of monoecious *S. cuneata* in the Red River Valley, Manitoba, in October 2013. Tubers were cleaned with 5% bleach solution to remove harmful organisms (Hunter-Cario 2007), rinsed with distilled water, and stored moist in Ziploc bags at 4 °C to induce dormancy (adapted from McIninch and Garbisch 1991). On May 24–25th, 2014, tubers were removed from cold storage, weighed, and planted 5–8 cm deep in individual, 12.7 × 12.7 cm square pots containing approximately 465 g of low-nutrient sediment composed of 1/3 loam and 2/3 sand. Pots were placed in water-filled coolers in the University of New Brunswick research greenhouse to break tuber dormancy. Three weeks after initial planting, 99% of tubers visibly germinated, and plants were selected from this stock based on health, and similarity in size. Ninety plants, remaining in original pots, were transferred to an outdoor facility and randomly placed in 20 L buckets evenly spaced on a level deck and covered by a 50% shade cloth to reduce heat exposure. Two air pumps continuously bubbled air through each bucket to keep CO_2_ and oxygen levels consistent.

Three trials were established: (1) nutrient-enriched water (with four treatments of varying dosages); (2) nutrient-enriched sediment (with four treatments of varying dosages), and (3) an unenriched control (SM Table 1). All buckets received 16 L of de-chlorinated municipal water. For the water trial, water was enriched with potassium phosphate and ammonium nitrate. For the sediment trials, nutrients were added as slow-release fertilizer wrapped in two layers of landscaping fabric to allow for permeability and ease of replacement, and buried below the sediment surface. Water in all buckets was refreshed biweekly, and buckets were scrubbed with 5% bleach solution and subsequently rinsed to reduce algal build up. Slow-release fertilizer packs were also changed biweekly. Plants were monitored daily, and all new emergent leaves were traced and digitized using a CanoScan LiDe 110 scanner to determine if leaf size and shape changed during the experiment. After a 10-week growing period, all plants were removed from pots and sediment was rinsed from roots before being separated into parts (i.e., roots, stems, tubers, and leaves), and weighed to determine aboveground (AG) and belowground (BG) biomass, and AG:BG biomass ratio. Tubers produced by each plant were counted and weighed.

**Table 1:** *Sagittaria cuneata* production among three nutrient trials at the termination of a 10-week nutrient enrichment experiment

Nutrient trial	Biomass (g)	AG mass (g)	BG mass (g)	AG:BG	Leaves per plant	Tubers per plant	Height (cm)
Control	2.39a (0.37)	0.90a (0.13)	1.40a (0.21)	0.53a (0.12)	0.7a (0.5)	0.9a (0.2)	20.4a (0.6)
Sediment	45.86b (5.66)	22.67b (2.89)	21.89b (2.99)	0.77b (0.07)	7.1b (0.7)	15.2b (1.5)	31.7b (1.4)
Water	3.66a (0.27)	1.56a (0.11)	2.04a (0.17)	0.55a (0.05)	1.7a (0.3)	2.1c (0.2)	23.7a (0.6)

Values presented as average (±SE), with different lower-case letters denoting significance (*p* < 0.05) among trials based on Tukey’s HSD test

*AG* aboveground, *BG* belowground, *AG:BG* aboveground to belowground biomass ratio

Water samples were collected on three occasions during the experiment and analyzed for total nitrogen (TNw) and phosphorus (TPw) at Environment Canada’s National Laboratory for Environmental Testing using standard methods (APHA 2005a, b). Sediment samples were collected at the end of the experiment, dried at 60 °C for 5 days, ground and sieved to 1 mm, stored frozen and later analyzed for Olsen-P (“Sed P”; Olsen et al. 1954), and nitrate and ammonium (“Sed N” for the sum of nitrate and ammonium; Keeney and Nelson 1982 ammonium; Willis and Gentry 1987 nitrate).

### Field study

Sampling was conducted along streams in the Red River Valley, Manitoba, Canada, in mid-August 2014. The Red River Valley spans about 13,000 km^2^ in southern Manitoba, with approximately 76% used for agricultural activities (Red River Basin Board 2000). It is characterized by a continental climate with the warmest and coldest months, on average, being July (20 °C) and January (−14.6 °C), respectively, and an average 427 mm of annual precipitation (1980–2010 records for Morden, MB; www.climate.weatheroffice.gc.ca). We selected 15 sites that exhibited variability in nutrient emitting activities in their catchments. Of these, 10 sites were ones previously described by Yates et al. (2012), who estimated the quantity of N and P produced by human activities (livestock, human population, cropland area) within subcatchments of the Red River using a principal component analysis (PCA). We selected an additional five study sites to fill gaps in the nutrient gradient. All 15 sites were on independent tributaries within the Red River Valley, with stream orders between 2 and 4.

*Sagittaria cuneata* plants were sampled along a 100 m reach using five, 900 cm^2^ quadrats distributed across the reach (adapted from Downing and Anderson 1985). Placement of quadrats was intended to be unbiased by blindly casting a quadrat downstream. Sampled plants were mature (i.e., flowering and/or bearing fruit) to facilitate species identification and present in at least 5 cm of water to ensure a potential influence of water chemistry. Only the three newest (or 2, if the plant had only 2 emergent leaves), fully formed emergent leaves were collected from each of three *S. cuneata* plants per quadrat (*n* = 42–45 per site; adapted from Cornelissen et al. 2003). Each leaf was scanned, weighed, placed in a standard plant press and dried at 40 °C for 5 days before being reweighed (note that four leaves were excluded due to improper drying). Water depth and height were measured at each plant. To quantify nutrients, one 500 mL grab water sample was collected at each site and sediment samples were collected from each quadrat (*n* = 5 per site) to a depth of 10 cm. Water sample were analyzed for TPw (APHA 2012) and TNw (USEPA 1993) at the University of Alberta. Sediment nutrient concentrations were analyzed as described previously. Light and air temperature loggers (HOBO Pendant Temperature and Light Data Logger) were placed at 11 of the 15 sites to determine average daylight (lux) and air temperature (°C) over the summer (May–August 2014) (SM Table 2).

**Table 2:** Results of three-way nested ANOVA testing the significant level of replication of leaf length and leaf shape (represented by scores of PC1) of emergent leaves of *Sagittaria cuneata* using the model: dependent variable = µ + site + quadrat (site) + plant (quadrat (site)) + ε, where 2–3 leaves were collected from three plants sampled in each of five quadrats at 15 sites (42–45 leaves per site, 658 total) in August 2014

Dependent variable	Source of variation	*df*	Mean square	*F*	*p*	Variance components		
							Estimate	%
√leaf length	Site	14	3.83	58.50	**<0.01**	$\sigma_{\text{S}}^{ 2}$	0.067	23
	Quad(Site)	60	0.99	15.10	**<0.01**	$\sigma_{\text{Q(S)}}^{2}$	0.076	26
	Plant((Quad)Site)	150	0.30	4.59	<**0.01**	$\sigma_{\text{P(Q(S))}}^{ 2}$	0.081	28
	Residual	432	0.07			$\sigma_{\text{e}}^{ 2}$	0.065	23
log_10_(PC1 + 0.5)	Site	14	0.85	37.52	<**0.01**	$\sigma_{\text{S}}^{ 2}$	0.017	30
	Quad(Site)	60	0.11	4.98	<**0.01**	$\sigma_{\text{Q(S)}}^{2}$	0.007	13
	Plant((Quad)Site)	150	0.05	2.21	<**0.01**	$\sigma_{\text{P(Q(S))}}^{ 2}$	0.009	16
	Residual	432	0.20			$\sigma_{\text{e}}^{ 2}$	0.023	41

Variance components were included for all random effects. Significance (*p* < 0.05) is indicated by bold font and variables were transformed as indicated to meet the assumptions of ANOVA

### Leaf Morphology

**Fig. 1:**
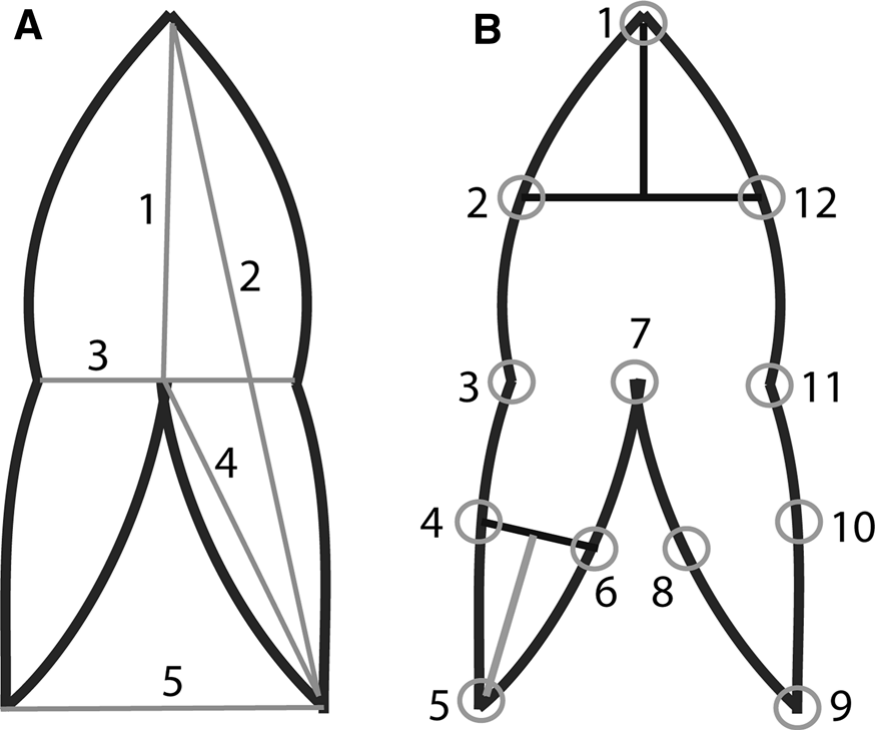
Leaf diagrams depicting **A** the six size measurements taken on each leaf: blade length (1), length (2), width (3), lobe length (4), distance between lobes (5), and the surface area of each leaf (not shown), and **B** the 12 landmarks chosen on each leaf to represent leaf shape, where landmarks 2 and 12 are exactly 50% of blade length and landmarks 4, 6, 8 and 10 are exactly 50% of lobe length All other landmarks are on fixed features of each leaf

To determine leaf size and shape, scanned leaf images from both studies were analyzed using ImageJ (Fig. 1; Rasband 1997–2014). Leaf size was denoted by six measurements (Fig. 1a). Differences in leaf shape were assessed using geometric morphometrics (Adams et al. 2004, 2012; Zelditch et al. 2004; Mitteroecker and Gunz 2009) based on 12 landmarks chosen to represent leaf shape (Fig. 1b). Euclidean coordinates were determined for each landmark on every leaf using ImageJ (Rasband 1997–2014).

### Statistical analyses

All statistical analyses were conducted using the statistical software, R (version 3.0.2, R Development Core Team, Vienna). One-way fixed-effects ANOVAs were used to assess differences in final plant height, number of leaves, and tubers produced by each plant, biomass (final, AG and BG), and AG:BG biomass ratio among experimental nutrient treatments. These analyses showed no significant differences (*p* > 0.05) of plant traits among treatments (i.e., dosages) within either the water-enriched or sediment-enriched trials. Therefore, results are presented for each trial (control, water-enriched, and sediment-enriched) with pooled treatments. When a significant main effect or interaction occurred, Tukey’s HSD tests were used to further assess trends in data.

Differences in leaf shape within both studies were assessed using geometric morphometrics. In R package *geomorph* v 1.1-6 (Adams and Otarola-Castillo 2013), a separate generalized Procrustes analysis (Gower 1975; Rohlf and Slice 1990) for each study was used to rotate and scale leaves thus removing size characteristics from data, resulting in 12 sets of scaled Euclidean coordinates (i.e., X and Y coordinates for landmarks 1, 2, 3, etc.) that represented the shape of individual leaves (henceforth called shape data). For each study, a principal components analysis (PCA) was conducted on shape data using R package *geomorph* v 1.1-6 (Adams and Otarola-Castillo 2013) to assess and compare individual leaf shapes. Principal components (PC) 1 and 2 were examined to ensure that each showed a meaningful shape gradient. PC1 was extracted for the field study PCA and used as a response in mixed linear effects models.

For the field study, Pearson correlations were calculated to determine relationships among leaf size characteristics, and between plant height and leaf size measurements, as well as water depth. To test for significant variation in leaf size and shape within levels of replication [leaf (two or three replicates per plant), plant (three replicates per quadrat), quadrat (five replicates per site), site (15 sites)], we conducted a three-way nested ANOVAs (Underwood 1997) to test the model: dependent variable = µ + Site + Quadat(Site) + Plant(Quadrat(Site)) + ε. Variance components were estimated for all random factors in each model using R package *lme4* (Bates et al. 2014) to determine at which scale the majority of random variation of leaf traits occurred.

To determine influences of nutrients on leaf morphology, nine linear mixed effects (LME) models predicting leaf traits were developed a priori based on known relationships of nutrients (water and sediment) and water depth with macrophyte growth: positive influences of TPw and TNw, singly and in combination; positive influences of SedP and SedN, singly and in combination; positive influences of both water and sediment nutrients; negative influence of water depth; and a global model with a positive influence of nutrients (water and sediment) and a negative influence of water depth. We included a random term (1| site/quadrat/plant) to account for variation at each level of replication, and a null model to determine whether random chance and/or variables outside of this study were influencing leaf traits. After assessment of a priori models, post hoc models were developed to explore the influence of additional variables (temperature, pH, latitude, longitude, Sed NO_3_) on leaf size of *S. cuneata*. LME models were calculated using R package *lme4* (Bates et al. 2014).

To determine which model was the “best” fit, the corrected Akaike Information Criterion (AICc; Sugiura 1978; Hurvich and Tsai 1991) was calculated for each model using R package *AICcmodavg* (Mazerolle 2015). The model with the lowest AICc value was deemed “best”, and models with a change in AICc (i.e., Δ_i_) of less than 4 were considered plausible (i.e., competing models). Models with Δ_i_ of 4–7 were considered less plausible, and values over seven were determined as implausible (as suggested by Anderson 2008).

## Results

### Experimental study

Comparison of plant traits among the three trials showed that plants grown in nutrient-enriched sediments were more productive than water-enriched and control trials (Table 1). Of plants in the sediment trial, 87 and 100% produced emergent leaves and tubers, respectively. In contrast, 65 and 98% of water trial and 20% and 70% of control trial plants produced emergent leaves and tubers, respectively. Final biomass (*F*_2,77_ = 102.56), BG (*F*_2,77_ = 85.86) and AG biomass (*F*_2,77_ = 101.71), AG:BG biomass ratio (*F*_2,77_ = 5.92), final plant height (*F*_2,77_ = 5.62), and both number of leaves (*F*_2,77_ = 25.64) and number of tubers (*F*_2,77_ = 101.96) per plant were significantly different (*p* < 0.01 for all comparisons) among the three nutrient trials, with sediment trial plants having higher biomass (final, AG and BG), larger AG:BG biomass ratio, greater height, and more leaves and tubers than water and control trials (*p* < 0.05, Tukey’s HSD test; Table 1). In addition, water trial plants produced 2.5X more emergent leaves and had significantly more tubers per plant than control trial plants.

**Fig. 2:**
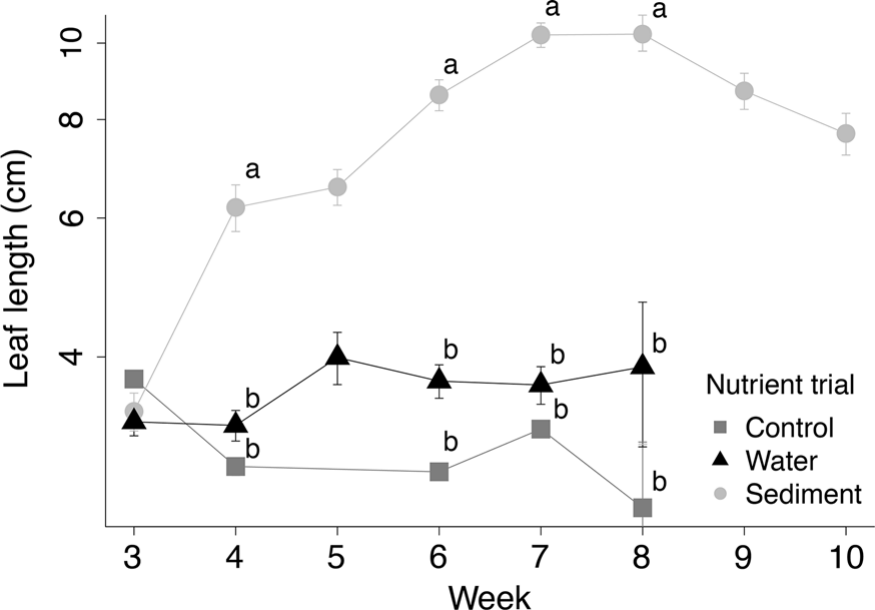
Average length (±SE; on a logarithmic scale) of emergent *Sagittaria cuneata* leaves (n = 2–40 for each trial and week, total = 272 leaves). Plants were grown in 1 of 3 different nutrient trials during a 10-week nutrient addition experiment. Different letters for a given week denote significance (*p* < 0.05, Tukey’s HSD test) among trials. Plants began producing emergent leaves in week 3; water-enriched and/or control plants did not produce emergent leaves in weeks 5, 9 and 10

Leaf length was chosen to represent leaf size because it had strong (*r* > 0.80), significant (*p* < 0.05) correlations with all other size characteristics (Fig. 1a). Leaf length was significantly influenced by an interaction between nutrient trial and weeks (*F*_*8,172*_ = 9.03, *p* < 0.01). Leaf length did not differ (*p* > 0.10, Tukey’s HSD test) among the 3 trials during the first week in which emergent leaves were present (i.e., week 3); however, during subsequent weeks, plants in the sediment trial produced larger emergent leaves than those grown in either the water or control trials (Fig. 2). In the case of the sediment trial, leaves were smallest during week 3, largest during weeks 7 and 8, and intermediate and similar in size during weeks 4, 5, 6, and 10. For plants in the water and control trials, emergent leaves remained small and similar in size throughout the experiment, and were not produced in weeks 9 and 10.

**Fig. 3:**
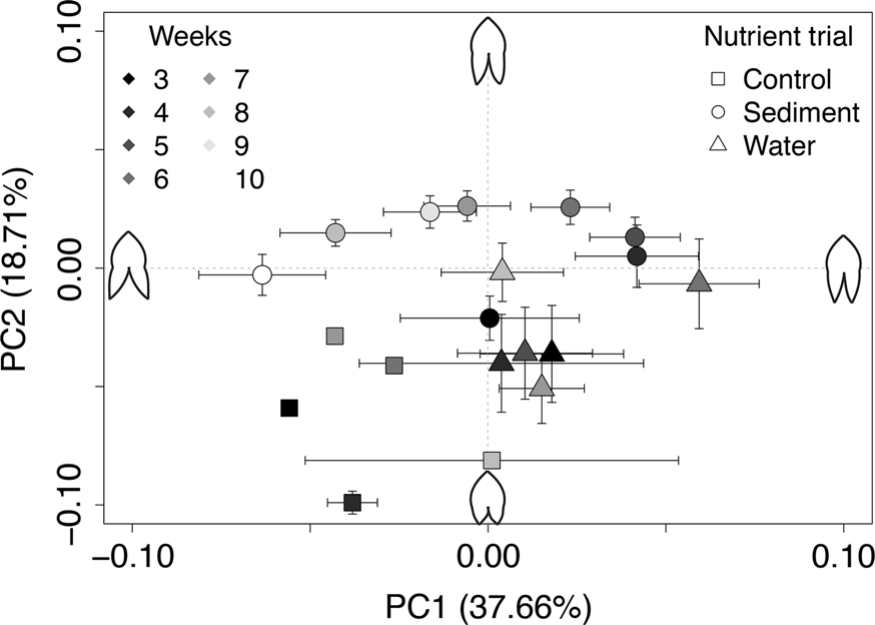
Bivariate plot of average (±SE) principal components (PC) 1 and 2 scores depicting variation in landmark coordinates (i.e., shape) of emergent *Sagittaria cuneata* leaves (n = 2–40 for each trial per week, total = 272 leaves) grown in three nutrient trials during a 10-week experiment Leaves began emerging in week 3 of the experiment. Line drawings illustrate the change in leaf shape depicted by each axis, with PC 1 associated with a change in lobe distance (Fig. 1b, landmark 5 and 9) and PC 2 indicative of a change in lobe length (Fig. 1b, landmark 5 and 7, or 9 and 7)

Similar to leaf length, shape of emergent *S. cuneata* leaves varied among the three trials and over time (Fig. 3). About 57% of shape variation was explained by gradients associated with PC 1 and PC 2, which corresponded to the distance between lobes (variation between landmarks 5 and 9; Fig. 1b) and lobe length (variation between landmarks 5 and 7, or 9 and 7; Fig. 1b), respectively. Leaves produced in the control trial were of a similar shape (Fig. 3), though the control trial had low replication of emergent leaves (n = 7). Similar to control plants, the average shape of water-trial leaves showed little variation over the experiment. In contrast, average leaf shape of the sediment trial changed weekly. On average, leaves produced by plants in the sediment trial also had longer lobes compared to those produced in the water and control trials.

### Field study

**Fig. 4:**
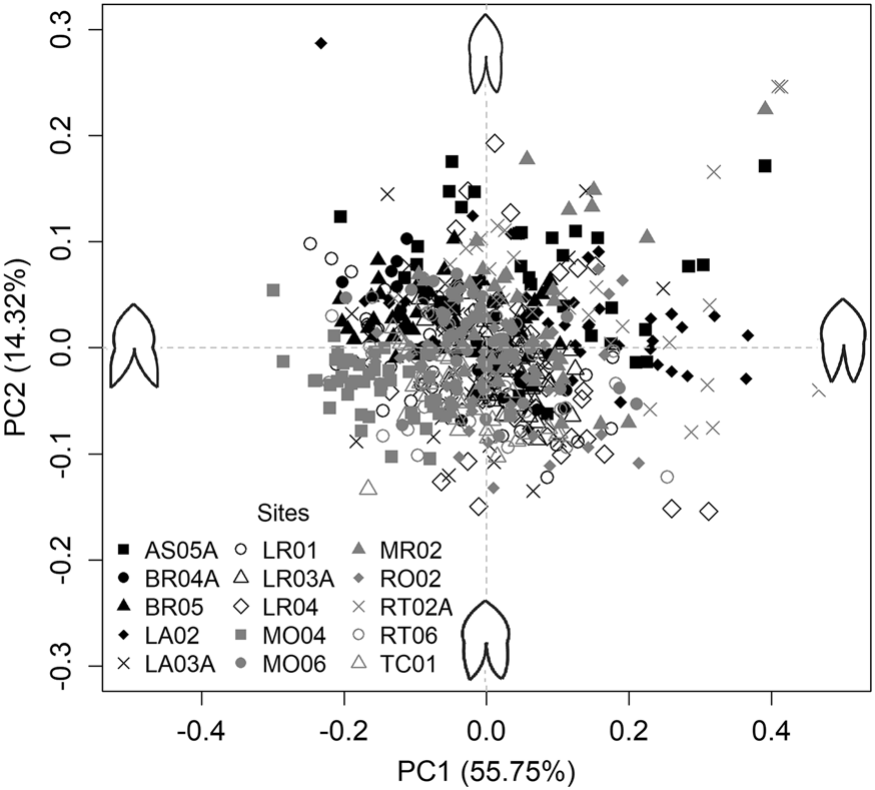
Bivariate plot of unaveraged (n = 42–45 leaves per site, 658 total leaves) principal components (PC) 1 and 2 scores summarizing variation in 12 sets of Euclidean landmark coordinates (i.e., shape, see Fig. 3) of *Sagittaria cuneata* leaves collected from 15 tributary sites in the Red River Valley, Manitoba, Canada, in August 2014

Principal components analysis (PCA) showed that leaf shape was variable within and among sites, with little distinct grouping (Fig. 4). Principal component (PC) 1 explained about 56% of leaf shape variation and corresponded to differences in the rotation of the lobes, whereas PC 2 explained 14% of shape variation and related to width of leaf blades. Three-way nested ANOVA of leaf size also showed that leaf length varied significantly at site, quadrat, and plant replication scales; variance components for all random effects indicated that variation was similar at all four scales (about 25%; Table 2). In contrast, nested ANOVA of leaf shape (represented by scores of PC1) showed greatest variation at the leaf scale (41%), followed by site (30%) and quadrat (13%) and plant (16%) scale.

Comparison of a priori linear mixed effects models using AICc indicated that, for leaf length (Table 3) and leaf shape (Table 4), the null model was the “best” model. For leaf shape, all other models were greater than 9 AICc units from the null model, suggesting that none of the variables, singly or in combination, were influencing leaf shape of *S. cuneata*. For leaf length, however, TNw is considered a plausible model with a Δ_i_ of 2.55 units and a weight of 0.2, though the weight of the null model was 3.5× times greater (0.71). The R^2^ values show that the fixed factor of the model (TNw) explained about 10% of variation in leaf length, and the fixed and random terms combined explained 77% of variation.

**Table 3:** Comparison of a priori and post hoc models for predicting (a) leaf length and (b) leaf shape of emergent *Sagittaria cuneata* leaves in 15 streams of the Red River Valley, Manitoba, Canada in August 2014, using linear mixed effects (LME) models and corrected Akaike Information Criterion (AICc)

Model #	Model variables	$R_{M}^{2}$	$R_{C}^{2}$	AIC_c_	Δ_i_	Likelihood	ω_i_
A priori							
	Null model	0	0.7654	541.95	0	1.000	0.710
2	TNw	0.1037	0.7712	544.50	2.55	0.279	0.198
1	TPw	0.0498	0.7738	548.04	6.09	0.048	0.034
7	TNw, TPw	0.1086	0.7745	548.49	6.54	0.038	0.027
4	√Sed N	0.0134	0.7749	549.93	7.98	0.018	0.013
5	√depth	0.0074	0.7766	550.52	8.57	0.014	0.010
3	√Sed P	0.0034	0.7721	551.11	9.16	0.010	0.007
6	√Sed P, √Sed N	0.0153	0.7769	556.00	14.05	0.001	0.001
8	√Sed P, √Sed N, TNw, TPw	0.1086	0.7810	560.59	18.64	0.000	0.000
9	Sed P, Sed N, TNw, TPw, depth	0.1170	0.7855	564.79	22.84	0.000	0.000
Post hoc							
	Null model	0	0.7654	541.95	0	1	0.960
1	Sed NO_3_, TNw	0.1084	0.7724	550.11	8.16	0.017	0.016
5	Sed NO_3_	0.0069	0.7705	550.68	8.73	0.013	0.012
7	longitude, latitude	0.0548	0.7782	552.92	10.97	0.004	0.004
2	Sed NO_3_, TNw, TPw	0.1135	0.7754	554.07	12.12	0.002	0.002
4	pH, Temp_w_	0.1308	0.7804	555.70	13.75	0.001	0.001
8	Sed NO_3_, Temp_w_	0.0077	0.7757	555.94	13.99	0.001	0.001
3	√Sed P, √Sed N	0.0153	0.7769	556.00	14.05	0.001	0.001
6	Sed NO_3_, pH	0.0064	0.7757	556.02	14.07	0.001	0.001
9	Sed NO_3_, √Sed P	0.0103	0.7730	556.60	14.65	0.001	0.001
17	Sed NO_3_, TNw, TPw, latitude	0.1338	0.7763	557.89	15.94	0.000	0.000
16	Sed NO_3_, TNw, TPw, latitude, Temp_w_	0.1828	0.7758	559.03	17.08	0.000	0.000
10	Sed NO_3_, TNw, latitude, Temp_w_, pH	0.1872	0.7755	560.02	18.07	0.000	0.000
11	Sed NO_3_, pH, Temp_w_	0.0073	0.7814	561.17	19.22	0.000	0.000
14	Sed NO_3_, TNw, TPw, latitude, longitude	0.1308	0.7804	563.12	21.17	0.000	0.000
12	Sed NO_3_, TNw, TPw, latitude, √Sed P	0.1338	0.7763	564.12	22.17	0.000	0.000
15	Sed NO_3_, TNw, TPw, latitude, Temp_w_, pH	0.1841	0.7788	564.68	22.73	0.000	0.000
13	Sed NO_3_, TNw, latitude, Temp_w_, pH, √depth	0.1848	0.7782	566.08	24.13	0.000	0.000
	Global model	0.1762	0.7892	581.70	39.75	0.000	0.000

Variables are transformed as indicated to meet assumptions of LME. Also presented are the marginal R^2^ ($R_{M}^{2}$) and conditional R^2^ ($R_{C}^{2}$) values, change in AIC_c_ (Δ_i_), relative model likelihood, and model weight (ω_i_)

*Sed P* bioavailable phosphorus in sediment in the form of Olsen-P, *Sed N* nitrate and ammonia in sediment, *Sed NO*_*3*_ nitrate in sediment, *TNw* total nitrogen in water, *TPw* total phosphorus in water, *Temp*_*w*_ water temperature

**Table 4:** Comparison of a priori and post hoc models for predicting leaf shape of emergent *Sagittaria cuneata* leaves in 15 streams of the Red River Valley, Manitoba, Canada in August 2014, using linear mixed effects (LME) models and corrected Akaike Information Criterion (AICc)

Model #	Model variables	$R_{M}^{2}$	$R_{C}^{2}$	AIC_c_	Δ_i_	Likelihood	ω_i_
A priori							
	Null model	0.00	0.60	−1260.23	0.00	1.000	0.986
2	TNw	0.09	0.61	−1250.41	9.82	0.007	0.007
1	TPw	0.07	0.61	−1249.09	11.14	0.004	0.004
5	√depth	0.03	0.63	−1247.96	12.27	0.002	0.002
4	√Sed N	0.00	0.61	−1244.51	15.72	0.000	0.000
3	√Sed P	0.00	0.61	−1244.31	15.92	0.000	0.000
7	TNw, TPw	0.09	0.62	−1243.02	17.21	0.000	0.000
6	√Sed P, √Sed N	0.00	0.61	−1234.93	25.30	0.000	0.000
8	√Sed P, √Sed N, TNw, TPw	0.09	0.62	−1223.87	36.36	0.000	0.000
9	Sed P, Sed N, TNw, TPw, depth	0.11	0.64	−1218.12	42.11	0.000	0.000
Post hoc							
	Null model	0.00	0.60	−1260.23	0.00	1.000	1.000
1	Sed NO_3_, TNw	0.09	0.61	−1240.78	19.45	0.000	0.000
2	Sed NO_3_, TNw, TPw	0.09	0.62	−1233.40	26.83	0.000	0.000
5	Sed NO_3_	0.00	0.61	−1244.36	15.87	0.000	0.000
10	Sed NO_3_, TNw, latitude, Temp_w_, pH	0.09	0.64	−1216.23	44.00	0.000	0.000
7	longitude, latitude	0.01	0.63	−1237.82	22.41	0.000	0.000
16	Sed NO_3_, TNw, TPw, latitude, Temp_w_	0.09	0.64	−1217.59	42.64	0.000	0.000
17	Sed NO_3_, TNw, TPw, latitude	0.09	0.63	−1225.09	35.14	0.000	0.000
4	pH, Temp_w_	0.01	0.63	−1237.82	22.41	0.000	0.000
3	√Sed P, √Sed N	0.00	0.61	−1234.93	25.30	0.000	0.000
6	Sed NO_3_, pH	0.01	0.62	−1236.30	23.93	0.000	0.000
8	Sed NO_3_, Temp_w_	0.01	0.62	−1236.35	23.88	0.000	0.000
9	Sed NO_3_, √Sed P	0.00	0.61	−1234.76	25.47	0.000	0.000
15	Sed NO_3_, TNw, TPw, latitude, Temp_w_, pH	0.09	0.65	−1209.43	50.80	0.000	0.000
13	Sed NO_3_, TNw, latitude, Temp_w_, pH, √depth	0.11	0.66	−1210.35	49.88	0.000	0.000
14	Sed NO_3_, TNw, TPw, latitude, longitude	0.14	0.63	−1220.37	39.86	0.000	0.000
12	Sed NO_3_, TNw, TPw, latitude, √Sed P	0.09	0.63	−1215.49	44.74	0.000	0.000
11	Sed NO_3_, pH, Temp_w_	0.01	0.63	−1228.29	31.94	0.000	0.000
	Global model	0.19	0.64	−1193.00	67.23	0.000	0.000

Variables are transformed as indicated to meet assumptions of LME. Also presented are the marginal R^2^ ($R_{M}^{2}$) and conditional R^2^ ($R_{C}^{2}$) values, change in AIC_c_ (Δ_i_), relative model likelihood, and model weight (ω_i_)

*Sed P* bioavailable phosphorus in sediment in the form of Olsen-P, *Sed N* nitrate and ammonia in sediment, *Sed NO*_*3*_ nitrate in sediment, *TNw* total nitrogen in water, *TPw* total phosphorus in water, *Temp*_*w*_ water temperature

Similarly, post hoc comparisons showed that the null model was the best model for both leaf length (Table 3) and leaf shape (Table 4), suggesting that variables not considered in this study or random variation are influencing leaf traits. All other models were considered not plausible, based on Δi values greater than 7.

## Discussion

When the emergent plant *Sagittaria cuneata* was grown under controlled conditions, extreme differences were observed between plants growing in nutrient-enriched sediment and those propagated in nutrient-enriched water or unamended control conditions. Plants grown in nutrient-enriched sediments were much more productive. In contrast, plants in nutrient-enriched water trial were indistinguishable from those in the control in leaf size, plant height, and biomass, differing only in the fact that water-enriched plants produced more tubers and leaves than control plants. Many submerged aquatic macrophytes preferentially utilize sediment nutrients over water nutrients (Nichols and Keeney 1976a, b; Best and Mantai 1978; Carignan and Kalff 1980; Barko and Smart 1980, 1981, 1986; Chambers and Kalff 1985; Smith and Adams 1986; Madsen et al. 2001), although highly enriched water can sometimes offset the effects of low nutrient sediments (e.g., Rattray et al. 1991). Our observations that sediment trial plants were more productive and had higher AG:BG ratio than either water or control trial plants indicate that the emergent *S. cuneata* was accessing and utilizing sediment nutrient sources. Plants in the control trial grew poorly and often remained in submerged form, indicating that they did not have access to sufficient nutrients to reach maturity and thus produce emergent leaves and numerous reproductive propagules. The finding that AG:BG biomass ratios were greater for the sediment-enriched plants compared to both the water-enriched and control trials is consistent with our hypothesis of nutrient insufficiency in the latter two trials, as both terrestrial and aquatic plants allocate resources to belowground production instead of aboveground structures (Neill 1990; Hossain et al. 2004; Ket et al. 2011) when nutrient availability is limited (Darby and Turner 2008).

In addition to differences in productivity in response to nutrient enrichment, emergent leaf morphology varied among the three experimental trials. Leaf size of other species of *Sagittaria*, as well as various terrestrial plant species (Medina 1970; Atkinson 1973; Gulmon and Chu 1981; Jurik et al. 1982), has been shown to respond to nutrient availability. For example, leaf size of *S. latifolia* and blade width of *S. lancifolia* leaves were found to be larger in higher nutrient environments (Dorken and Barrett 2004, Richards and Ivey 2004), consistent with our experimental observations. Wooten (1986) reported a decrease in leaf size with increasing water depth for several *Sagittaria* spp.; however, water depth was, on average, consistent among our nutrient treatments and thus unlikely to be influencing leaf size. Rather, nutrients added to sediments were responsible for the increased leaf size (and greater productivity) of sediment trial plants. In addition, leaf shape of *Sagittaria* has been shown to vary with nutrient availability: leaf shape of monoecious and dioecious *S. latifolia* plants changed based on resource availability and leaf age, producing thinner leaves when grown in high compared to low fertilizer treatments and with more pronounced differences later in the growth cycle (Dorken and Barrett 2004). Our experimental study also found differences in leaf shape in response to nutrient-enrichment and with plant maturation, with *S. cuneata* plants in the sediment trial producing leaves with lobes that were close together initially that later changed to leaves with lobes that were further apart. Availability of nutrients for plant uptake has far-reaching implications: leaves of varying shape (i.e., Nicotra et al. 2011) and larger size increase photosynthetic area (Parkhurst and Loucks 1972; Jurik et al. 1982; Niinemets and Kull 1994) and, hence, energy accrual which, in turn, is manifest in greater clonal (i.e., tuber production) or sexual reproduction (i.e., flower and seed production) (Bazzaz et al. 1987; Reekie and Bazzaz 1987). In the case of *S. cuneata*, leaf size began to decrease near the end of the growing season (weeks 9 and 10), and leaf shape reverted to earlier shapes when plants were allocating nutrients to production of over-wintering propagules. In fact, upon harvesting sediment trial plants were found to have 6.4 and 15.2X more tubers than the water and control trials, respectively, thus ensuring a greater likelihood of survival and regeneration during the following year.

Leaf morphology from the field study was markedly different from that of the experimental study. Rather than the strong response to sediment nutrients shown by experimental plants, field collected plants exhibited extreme variability in leaf morphology that was not explained by sediment nutrients or multiple other abiotic variables. Instead, random chance (or variables such as water velocity and turbidity that were not included in our field study but held constant in the experimental study) may have strongly influenced leaf morphology of *S. cuneata* under natural conditions. Results of nested ANOVAs of leaf length and shape suggest that leaf morphology is governed by complicated processes operating at a variety of scales: leaf, plant, quadrat and site scale, such that leaf length exhibited similar variance at all four scales whereas leaf shape was most variable at extreme scales (leaf and site). Site scale variation is likely related to the genetics of the population since *S. cuneata* generally reproduces clonally, as do most macrophytes (Jones et al. 2012); thus, plants within a site are more genetically similar than plants from different sites. Variation in leaf traits undoubtedly also occurs because of environmental heterogeneity (quadrat scale); differences in plant age, resource availability, and the competitive interaction for resources amongst plants of different age (plant scale); and within plant variation of leaf age (leaf scale). To our knowledge, there are no studies that have quantified patterns of variation in leaf characteristics within and among *Sagittaria cuneata* populations. However, in the case of terrestrial plants, Bruschi et al. (2003) observed that for the sessile oak *Quercus petraea*, morphological traits such as leaf length and leaf width varied within a single tree, among trees in a single population, and among populations, with the variance in morphological traits being almost equal (14–20%) among these three scales. Collectively, these findings emphasize the need to standardize sampling design when collecting leaves for autecological and taxonomic studies. This is particularly true for aquatic macrophytes as many species (such as *S. cuneata*) exhibit highly variable leaf plasticity.

Collectively, the experimental and field studies show that leaf development of *Sagittaria cuneata* is a complicated process: under controlled settings, sediment nutrients drive plant growth whereas under field conditions, leaf morphology is highly variable at all hierarchical scales, with no over-arching environmental drivers. Though results of both studies provide new information on the life history of *S. cuneata* and the relationship between leaf morphology and its surrounding environment, the variability of leaf morphology in the field proves difficult for use as a bioindicator. A good bioindicator should show greatest variability at the site scale, with the trait or metric showing a dose response to the environmental stressor. However, the field study demonstrated relatively equal variability of leaf traits among the leaf, plant, quadrat and site scales, suggesting large natural variability within the species, populations, and individuals. *Sagittaria cuneata* is currently not a reliable indicator of stream nutrients, though further in situ study could tease apart the influences of stream variables and characteristics on leaf morphology of *S. cuneata*. Our results do, however, emphasize the need to quantify within and among plant variation in leaf morphology, especially for aquatic macrophytes. The latter represent a diverse group of aquatic photosynthetic organisms with leaf (or frond) size ranging from <0.5 mm to 2.5 m (Chambers et al. 2008) that are often identified based on growth form and foliage characteristics. Sampling methods that do not account for high variation in leaf morphology may exacerbate the already notorious difficulty in classifying many taxa of aquatic plants.

## Electronic supplementary material

Below is the link to the electronic supplementary material.

Supplementary material 1 (DOCX 64 kb)
