# Supplementary material for: Arrowhead (Sagittaria cuneata) as a bioindicator of nitrogen and phosphorus for prairie streams and wetlands
Source: Wetl Ecol Manag. 2017 Sep 27;26(3):331–43. doi: 10.1007/s11273-017-9576-5 (PMC6438639; doi:10.1007/s11273-017-9576-5)
Supplement: Supplementary file 1 — Supplementary material 1 (DOCX 64 kb) [file 11273_2017_9576_MOESM1_ESM.docx]

**Supplementary Material**

Table 1. Fertilizer amount and type added every two weeks to either low-nutrient water (4 treatments) or low-nutrient sediment (4 treatments), in addition to a control where nutrients were not added. Also shown are measured sediment (Sed N and Sed P) and water (TN_w_ and TP_w_) nutrient concentrations, and water quality measurements (mean ± SE). Sed P = sediment phosphorus as Olsen-P; Sed N = sediment nitrogen as nitrate + ammoniun; TPw = total phosphorus in water; TNw = total nitrogen in water; SPC = specific conductivity; DO = dissolved oxygen; Depth = water depth.

| Trial  Treatment | Fertilizer Additions |  | Sed N (mg/kg) | Sed P  (mg/kg) | TN_w_ (mg/L) | TP_w_ (mg/L) | DO (%) | SPC (µS/cm) | Turbidity (NTU) | pH | Depth (cm) |
| --- | --- | --- | --- | --- | --- | --- | --- | --- | --- | --- | --- |
| Control | – |  | 17.35 (2.25) | 8.02  (1.35) | 0.15 (0.02) | 0.01 (0.00) | 101  (1) | 265  (4) | 2.6  (0.5) | 8.5 (0.0) | 27.6 (0.3) |
| Sediment |  |  |  |  |  |  |  |  |  |  |  |
| HP,HN | 15 g fertilizer^1^ |  | 50.38 (8.66) | 174.67 (36.83) | 1.11 (0.34) | 0.24 (0.10) | 110  (2) | 247  (5) | 9.3  (1.0) | 8.7 (0.2) | 27.5 (0.2) |
| HP,LN | 15 g fertilizer^2^  + 3 g bonemeal |  | 72.98 (6.51) | 37.60  (3.20) | 1.11 (0.49) | 0.08 (0.02) | 104  (1) | 269  (4) | 8.4  (1.3) | 8.7 (0.0) | 27.6 (0.3) |
| LP,HN | 6 g fertilizer^3^ |  | 239.16 (93.37) | 67.00 (43.64) | 6.77 (3.00) | 0.06 (0.04) | 105  (2) | 331  (12) | 8.0  (1.8) | 8.5 (0.1) | 27.1 (0.4) |
| LP,LN | 5 g fertilizer^1^ |  | 13.41 (1.38) | 10.06  (1.85) | 0.42 (0.09) | 0.03 (0.01) | 103  (1) | 264  (4) | 4.9  (0.6) | 8.7 (0.0) | 27.7 (0.2) |
| Water |  |  |  |  |  |  |  |  |  |  |  |
| HP,HN | 0.095 g KH_2_PO_4_; 0.123 g NH_4_NO_3_ |  | 15.98 (3.35) | 9.32  (0.86) | 1.30 (0.35) | 0.60 (0.21) | 111  (2) | 231  (4) | 3.9  (0.5) | 9.1 (0.1) | 27.3 (0.3) |
| HP,LN | 0.095 g KH_2_PO_4_; 0.025 g NH_4_NO_3_ |  | 14.87 (1.49) | 8.81  (1.14) | 0.78 (0.10) | 0.64 (0.21) | 107  (1) | 251  (4) | 2.6  (0.6) | 8.8 (0.1) | 27.3 (0.3) |
| LP,HN | 0.003 g KH_2_PO_4_; 0.123 g NH_4_NO_3_ |  | 14.81 (1.16) | 8.43  (0.72) | 1.65 (0.31) | 0.02 (0.00) | 103  (1) | 240  (5) | 4.1  (0.6) | 8.6 (0.0) | 27.0 (0.2) |
| LP,LN | 0.003 g KH_2_PO_4_; 0025 g NH_4_NO_3_ |  | 13.38 (0.38) | 8.62  (0.67) | 0.38 (0.05) | 0.02 (0.00) | 102  (1) | 250  (4) | 4.6  (2.0) | 8.6 (0.2) | 27.0 (0.2) |

^1^Plant-Prod Smartcote (14-14-14; NPK); ^2^Miracle-Gro Shake ‘n Feed (9-18-9); ^3^Miracle-Gro Shake ‘n Feed (12-4-8)

Supplementary Material Table 2. Water quality and sediment nutrients at 15 tributary sites in the Red River valley in southern Manitoba, Canada, in August 2014. Sed P = sediment phosphorus as Olsen-P (average ± SE); Sed N = sediment nitrogen as nitrate + ammonium (average ± SE); TPw = total phosphorus in water; TNw = total nitrogen in water; pH, specific conductivity (SPC); DO = dissolved oxygen; Temp = water temperature; Depth = water depth, air temperature and light during daylight hours are presented. Blank spaces represent data missing for these variables due to equipment failure or absent loggers.

| Sediment | | Water | | | | | | |  |  |  |  |
| --- | --- | --- | --- | --- | --- | --- | --- | --- | --- | --- | --- | --- |
| Site | Sed P (mg P/kg) | Sed N (mg N/kg) | TPw (mg/L) | TNw (mg/L) | pH | SPC (µS/cm) | DO (%) | Temp  (°C) | Depth (cm) | Air Temp. (°C) | Light (lux) | |
| AS05A | 50.24 (19.70) | 70.71 (3.88) | 1.35 | 2.41 | 8.55 | 703 | 77 | 20 | 20.80 (1.44) | 22 (0.11) | 71 250 (950) | |
| BR04A | 13.78 (1.42) | 26.47 (0.35) | 0.12 | 1.08 | 8.26 | 508 | 54 | 21 | 11.93 (1.18) | 21 (0.11) | 56 500 (1052) | |
| BR05 | 23.04 (2.90) | 36.51 (1.94) | 0.12 | 1.21 | 8.46 | 517 | 89 | 21 | 12.73 (1.10) | 22 (0.11) | 72 500 (1006) | |
| LA02 | 30.90 (1.97) | 79.86 (3.93) | 2.74 | 2.51 | 8.01 | 837 |  | 16 | 40.67 (1.86) |  |  | |
| LA03A | 40.26 (7.73) | 83.47 (6.65) | 0.42 | 1.04 | 8.25 | 814 | 76 | 21 | 86.00 (2.33) | 21 (0.12) | 57 500 (998) | |
| LR01 | 11.29 (1.72) | 24.83 (1.77) | 0.08 | 1.01 | 8.57 | 505 | 96 | 23 | 30.25 (2.65) | 21 (0.11) | 74 000 (1085) | |
| LR03A | 32.28 (4.75) | 121.24 (5.77) | 0.04 | 1.38 | 8.13 | 599 | 86 | 18 | 36.80 (1.81) | 21 (0.11) | 69 500 (990) | |
| LR04 | 12.36 (0.94) | 57.20 (1.24) | 0.09 | 0.87 | 8.18 | 865 | 57 | 25 | 48.30 (1.85) | 21 (0.11) | 72 750 (1056) | |
| MO04 | 41.72 (2.83) | 66.28 (2.60) | 0.12 | 0.53 | 8.33 | 1021 | 74 | 19 | 31.87 (1.17) | 19 (0.06) | 6 500 (111) | |
| MO06 | 42.70 (2.85) | 67.97 (2.30) | 0.28 | 1.61 | 8.24 | 734 | 67 | 24 | 54.73 (5.13) | 22 (0.11) | 79 250 (1108) | |
| MR02 | 52.66 (3.64) | 86.70 (3.50) | 0.36 | 1.38 | 8.20 | 473 | 75 | 22 | 38.20 (2.02) |  |  | |
| RO02 | 26.06 (2.74) | 14.86 (2.22) | 0.04 | 1.33 | 8.27 | 556 | 85 | 21 | 22.13 (2.53) | 20 (0.09) | 45 250 (891) | |
| RT02A | 17.26 (1.16) | 14.31 (0.68) | 0.25 | 0.91 | 8.42 | 517 |  | 21 | 25.33 (3.84) |  |  | |
| RT06 | 31.44 (4.38) | 40.37 (2.06) | 0.15 | 1.19 | 8.18 | 388 | 73 | 21 | 15.27 (1.31) | 21 (0.10) | 67 500 (945) | |
| TC01 | 41.90 (4.80) | 64.99 (5.67) | 0.10 | 1.08 | 7.93 | 1290 | 71. | 22 | 64.80 (1.01) |  |  | |
